# Comparative evaluation of SARS-CoV-2 laboratory testing data sources for U.S. COVID-19 public health surveillance, 2020–2024

**DOI:** 10.3389/fpubh.2026.1827546

**Published:** 2026-06-23

**Authors:** Alisha Kalangara, Conor Millard, Amber K. Winn, Tricia A. Aden, Kim Del Guercio, Jourdan DeVies, Stephanie Dietz, Andrew Godoshian, Jason Hall, Teresa Jue, Joseph Miller, Erica Billig Rose, Zachary R. Smith, Benjamin J. Silk

**Affiliations:** 1 Goldbelt Professional Services, Chesapeake, VA, United States; 2 Coronavirus and Other Respiratory Viruses Division, National Center for Immunization and Respiratory Diseases, Centers for Disease Control and Prevention, Atlanta, GA, United States; 3 Division of Infectious Disease Readiness and Innovation, National Center for Emerging and Zoonotic Infectious Diseases, Centers for Disease Control and Prevention, Atlanta, GA, United States; 4 Deloitte Consulting LLP, Atlanta, GA, United States; 5 Office of Public Health Data, Surveillance, and Technology, Office of the Director, Centers for Disease Control and Prevention, Atlanta, GA, United States; 6 Office of Readiness and Response, Office of the Director, Centers for Disease Control and Prevention, Atlanta, GA, United States; 7 Office of Laboratory Systems and Response, Office of the Director, Centers for Disease Control and Prevention, Atlanta, GA, United States

**Keywords:** commercial laboratory, COVID-19 electronic laboratory reporting, COVID-19 response, increasing community access to testing, National Respiratory and Enteric Virus Surveillance System, SARS-CoV-2, surveillance

## Abstract

**Introduction:**

SARS-CoV-2 laboratory testing data were collected by the U.S. Centers for Disease Control and Prevention for surveillance during the COVID-19 pandemic response. Understanding how these existing data correlate and diverge can help guide their use and interpretation.

**Method:**

We evaluated COVID-19 Electronic Laboratory Reporting (CELR), the National Respiratory and Enteric Virus Surveillance System (NREVSS), Increasing Community Access to Testing, Treatment and Response (ICATT) program, and commercial laboratory data from 2020 to 2024. SARS-CoV-2 testing volume, viral activity, and viral activity trends were calculated for each source during periods marked by key events. Key events included Delta and Omicron variant emergence, at-home testing availability, and public health emergency conclusion. Viral activity was characterized by percent positivity, the median percent of tests positive for SARS-CoV-2. Viral activity trends were measured using Spearman’s correlation coefficients of normalized percent positivity between NREVSS and each of the data sources, along with match rates between the two sources from weekly trajectory categorization (increasing, stable, decreasing) comparisons.

**Results:**

SARS-CoV-2 testing volume decreased across time, most notably since January 2022. Viral activity measures showed that CELR (8.4%) and NREVSS (7.7%) had lower median weekly percent positivity compared to ICATT (18.5%) and commercial laboratories (11.0%). Viral activity trends between NREVSS and CELR (*ρ* = 0.93) and NREVSS and commercial laboratory (*ρ* = 0.85) were highly correlated, and less correlated between NREVSS and ICATT (*ρ* = 0.58). Week-to-week trajectory match rates indicated concordant viral trends across data sources with 89% for CELR, 79% for commercial laboratories, and 67% for ICATT when compared to NREVSS.

**Discussion:**

ICATT and commercial laboratory data sources consistently reported higher activity levels than CELR and NREVSS, suggesting differences in testing and care-seeking among the underlying populations represented, and the effects of varying reporting practices over time. However, trend correlations with NREVSS were high and few differences were observed in week-to-week trajectories. While these findings may be in part due to overlap in data sources, they also suggest that viral activity trends were relatively consistent across sources. A broad set of laboratory-based surveillance data sources effectively captured trends in SARS-CoV-2 circulation despite the evolving nature of testing and reporting during and after the COVID-19 pandemic.

## 1 Introduction

The public health response to the COVID-19 pandemic necessitated the development and expansion of timely surveillance systems that monitor trends in viral activity and allow for actionable public health responses (1, 2). In the United States, widespread laboratory testing was implemented, surveillance infrastructure was assembled, and several laboratory-based reporting systems and commercial laboratory networks collected SARS-CoV-2 testing data that were leveraged for surveillance. In March 2020, the Centers for Disease Control and Prevention (CDC) adapted existing electronic laboratory reporting infrastructure within jurisdictions and partner organizations to implement automated data feeds for receiving COVID-19 laboratory test results (3). Subsequently, surveillance data sources were affected by testing and reporting policy changes that occurred throughout the course of the pandemic (4). Notably, with the end of the public health emergency (PHE) declaration in May 2023, the U.S. Department of Health and Human Services (HHS) no longer required reporting of negative laboratory results through the COVID-19 electronic laboratory reporting (CELR) program (5). While CELR had previously been a robust source of SARS-CoV-2 laboratory testing data, it became necessary to examine other sources of SARS-CoV-2 laboratory testing data that continued to collect negative results to monitor percent positivity after the end of the PHE.

Understanding how data from various sources of laboratory testing correlate and differ over time can help guide the use and interpretation of U.S. COVID-19 laboratory-based surveillance data. This evaluation summarizes how national SARS-CoV-2 test volume stability, viral activity, and viral activity trends differed across data sources during key event periods expected to impact SARS-CoV-2 testing and reporting.

## 2 Methods

### 2.1 Data sources

For the timespan of March 2020 through May 2024, we consolidated four national sources of deidentified SARS-CoV-2 nucleic acid amplification test (NAAT) (including polymerase chain reaction, or PCR tests) data (Table 1). Across all datasets, only tests that were conclusively determined to be positive or negative were included and antigen tests were excluded. For this analysis, CELR was considered the most complete data set, as all diagnostic test results were mandated to be reported to CELR until the end of the PHE declaration on May 11, 2023. Due to some states’ reporting requirements, there were changes in reporting practices prior to this date. Additionally, 41 of 56 jurisdictions continued to send CELR data voluntarily after the end of the PHE and this gradually decreased until September 2024 when CELR was officially decommissioned at CDC. For the purpose of this evaluation, however, CELR data after the end of the PHE were excluded (5, 6).

**Table 1:** Summary of U.S. laboratory testing data sources for COVID-19 public health surveillance, 2020–2024.

Data source attribute	COVID-19 electronic laboratory reporting (CELR)	National Respiratory and Enteric Virus Surveillance System (NREVSS)	Increasing Community Access to Testing, Treatment, and Response (ICATT)	Commercial laboratories
Source data availability period	March 2020–May 2023	March 2020–Present	April 2020–May 2025	March 2020–Present
Method of data collection	Extension of existing electronic laboratory reporting infrastructure for transmission of test results to state and local public health departments, healthcare systems, and CDC (6) Reporting of all COVID-19 testing to the CDC was mandated through the CELR system during the public health emergency (PHE) declaration (3, 5) Reporting times ranged from daily to weekly with an average lag of 7 days.	Passive, laboratory-based surveillance network Aggregates voluntarily reported data from >450 reporting laboratories in university and community hospitals, selected state and county public health departments, and commercial entities on a weekly basis (8)	Through commercial pharmacies and laboratory partners, provided no-cost COVID-19 testing for symptomatic or exposed individuals, and the uninsured (12) Supported COVID-19 testing for communities with less access to testing (11) Results are reported daily with a lag of up to 3 days	Data from two large commercial laboratories Laboratories report information on laboratory test orders and results via HL7 messaging to the National Syndromic Surveillance Program (NSSP) twice daily on most days (14)
Data quality and depth	Estimated to capture ~92% of COVID-19 testing occurring in the US (30)	Coverage across 50 U.S. states, Puerto Rico, and Washington, D.C. (10) Weekly reports of aggregate testing volume and positive specimen count without patient-level data (i.e., no individual-level information or demographics available)	Coverage across 50 U.S. states and DC and Puerto Rico (11) Captures individual- and specimen-level details, including demographics (age, sex, race, ethnicity), immunization status, symptoms, patient risk factors, and viral genomic data (11)	Coverage across 50 U.S. states and five territories Demographics (age, sex, race, and ethnicity) included as well as clinical information (e.g., setting of care) (14)
Data use notes	‘Gold standard’ for laboratory-based surveillance until the end of the PHE declaration (May 2023) Some reporters may also be included in both NREVSS and commercial laboratory data Incomplete reporting observed from some states prior to end of PHE	‘Gold standard’ for laboratory-based surveillance after end of PHE declaration (May 2023) Some reporters may be included in CELR and commercial laboratory data	May overrepresent COVID-19 positivity due to focus on socially vulnerable populations and individuals at high risk for SARS-Cov-2 infection	Some reporters may be included in NREVSS and CELR data

COVID-19 electronic laboratory reporting (CELR); National Respiratory and Enteric Virus Surveillance System (NREVSS); Increasing Community Access to Testing, Treatment, and Response (ICATT), public health emergency (PHE), centers for disease control and prevention (CDC), health level seven (HL7).

After the PHE, the National Respiratory and Enteric Virus Surveillance System (NREVSS) became CDC’s primary source of laboratory-based surveillance data for SARS-CoV-2 (7, 8). Since the 1980s, NREVSS has monitored seasonal trends in respiratory viral activity; SARS-CoV-2 testing data have been included since 2020 (9, 10). NREVSS collects weekly counts of testing volumes and positive results. Specimen data are reported in aggregate and do not include patient-specific information (e.g., demographics).

CDC’s Increasing Community Access to Testing, Treatment, and Response (ICATT) program does include patient-specific demographic, clinical, payer, geographical, and vaccination history data (11, 12). ICATT’s main objective was to support testing where access to testing is otherwise limited, and the program has supported socially vulnerable communities during periods of increased transmission. Since April 2020, ICATT has provided COVID-19 testing services at no-cost, through contracts with commercial pharmacies, for uninsured individuals with COVID-19 symptoms or suspected exposures (13). Along with these tests paid by ICATT, starting in early 2022 the ICATT data also included non-ICATT paid pharmacy tests (21% of total tests), which were covered using cash, private insurance, Medicare, or Medicaid.

Lastly, commercial laboratory testing data were obtained by contract from the National Syndromic Surveillance Program (NSSP) (14). This dataset consists of SARS-CoV-2 testing data from two large national commercial laboratories, which are reported in near real-time (i.e., a typical lag of <1 day between when results are received and reported). Like ICATT, the commercial laboratory testing data included demographic and clinical information, such as setting of care. For all sources, data collection and reporting were timely, occurring close to testing with a maximum lag of 1 week.

Of note, these data sources may share reported tests as tests reported to one source may have been reported to other sources. While overlap of sources is not ideal for this analysis, these data sources were chosen because they were used for surveillance during the pandemic.

We defined key event periods according to key events relevant to the COVID-19 pandemic. These events included the SARS-CoV-2 Delta variant attaining U.S. predominance (≥50% estimated prevalence) on June 1, 2021 (week ending date: 06/05/2021), the Omicron variant attaining U.S. predominance on December 19, 2021 (12/25/2021), at-home testing kits becoming available for order from the U.S. government on January 19, 2022 (01/22/2022), and the end of the COVID-19 PHE (5, 15–18). Data were downloaded and finalized for analysis by August 2025. These were aggregated to the weekly level using MMWR (epidemiologic) weeks (19).

### 2.2 Stability, viral activity, correlations, and trajectory matches

We developed four complementary measures to compare test volume stability, viral activity level, and correlations and trajectory matches with viral activity trends among the data sources. We also estimate sample overlap between sources, noted as the percentage of tests estimated to be reported to multiple data sources.

To compare test volume stability, the overall weekly maximum testing volume was determined for each source. Additionally, the number of health service areas (HSAs) that each data source covered per month was assessed. For a HSA to be included in the month, the data source must have received >10 tests from the HSA during that month. This threshold is based on the National Center for Health Statistics standard for counts (20). The median and interquartile range (IQR) for monthly number of HSAs covered was calculated and used to determine the coefficient of quartile variation (IQR/median) for each data source to quantify variability. The median and IQR were selected as the data appeared left skewed, and these measures are less susceptible to outliers. The coefficient of quartile variation (CQV) was used as the IQR itself is scale-dependent and this measure normalizes these values. Smaller coefficients indicate more stability as the spread of reporters compared to the typical level of reporting is small.

As an indicator of viral activity, weekly percent positivity measures were calculated for each data source by dividing the number of positive tests during a week by the weekly testing volume (and multiplying by 100). To compare differences in viral activity, the median of weekly percent positivity was calculated by data source for each key event period. A Wilcoxon signed-rank test was conducted overall and for each key event period to compare NREVSS with the other data sources.

To compare viral activity trends across data sources, correlations and trajectory matches were calculated for each data source, using NREVSS as the referent. NREVSS was selected as the referent system due to its historical and established role as the nationally recognized surveillance network for tracking trends in respiratory viruses. Furthermore, NREVSS continued laboratory-based data collection after the end of the PHE declaration and has maintained operational stability among reporters from participating laboratories. Because data sources had varying ranges for weekly percent positivity, each data source’s measures were normalized by rescaling the data between 0 and 1.0. By normalizing to the source specific maximum, the value of 1.0 indicated the highest weekly percent positivity across the entire time period for each data source. To examine the relative differences in normalized percent positivity (i.e., whether percent positivity peaks and troughs aligned across data sources), Spearman’s correlation coefficients were calculated in a comparison period when data were present for both data sources. Statistical significance of the coefficient was determined using *t*-tests run against a null of 0 (no correlation) and a significance level of *α* = 0.05. Normalizing to the maximum allows for easier interpretability compared to other less intuitive methods that we also examined (e.g., z-score, range, and log()); in a sensitivity analysis, these other normalization methods produced similar correlations coefficients. Because Spearman’s correlation is rank based, the strategies mentioned change the magnitude of weekly percent positivity but do not affect the correlation coefficient because the order of rank is not changed. Normalizing to the maximum is sensitive to outliers, notably when the peak is an outlier. This was not the case for the ICATT data, but does affect commercial, NREVSS, and CELR data in that normalized percent positivity values may appear lower and flatter compared to their peaks.

To characterize matches, weekly trajectories were defined using the standard error of a difference in percent positivity between a week and the previous week using the following.

$$SE_{p_{1}-p_{2}}=\sqrt{\frac{p_{1}(100-p_{1})}{n_{1}}+\frac{p_{2}(100-p_{2})}{n_{2}}}$$

where

$$p_{1}\;\text{and}\;p_{2}\;\mathrm{are}\;\text{percent positivity}\;\mathrm{at}\;\text{times}\;1\;\text{and}\;2$$

$$n_{1}\;\text{and}\;n_{2}\;\mathrm{are}\;\text{sample size}\;\mathrm{at}\;\text{times}\;1\;\text{and}\;2$$

Standard errors were used to construct a 95% confidence interval. The difference in percent positivity between two consecutive weeks was categorized as either stable (within 95% confidence interval) or not stable (outside the 95% confidence interval). Depending on whether the percent positivity was increasing or decreasing from the previous week, the non-stable weeks were further classified as either an increase or a decrease. This method is stable for sufficiently high testing volume using the following rule, which allows a normal distribution approximation.

n∗(100−p)≥500ANDn∗p≥500

where

𝑝 is percent positivity

nis sample size

When evaluating samples size as a function of percent positivity using the above rule, sample sizes were found to be sufficient for all weeks that data were present, across all data sources.

This method also assumes independence of the people being tested from week to week. This is not necessarily true for these data as repeat testers are not restricted from getting tested in consecutive weeks. Due to the structure of the data, it was not possible to identify repeat testers.

Categorizations were compared between NREVSS and each data source for the comparison period. Trajectory matches were defined as weeks where the categorizations from NREVSS and the other data sources were the same. Opposite trajectory mismatches were defined as weeks where one data source indicated increase, while the other indicated decrease. Stable trajectory mismatches were defined as weeks where one data source was stable while the other was either increasing or decreasing. Matching trajectory proportions were calculated by dividing the number of weeks that matched by the total number of weeks in the comparison period.

To estimate the sample overlap between CELR and other sources, source of testing categories present in the CELR dataset were manually grouped to best represent the source of testing for each of the other data sources. This grouping produced rough estimates of the degree of overlap or the percentage of tests that are thought to be shared between two data source. Quantification of sample overlap between NREVSS and ICATT and commercial data could not be estimated due to the structure of these data. All analyses were conducted using R software (version 4.1.1; R Foundation). This activity was reviewed by CDC, deemed not research, and was conducted consistent with applicable federal law and CDC policy.^1^

## 3 Results

### 3.1 Test volume stability

**Figure 1:**
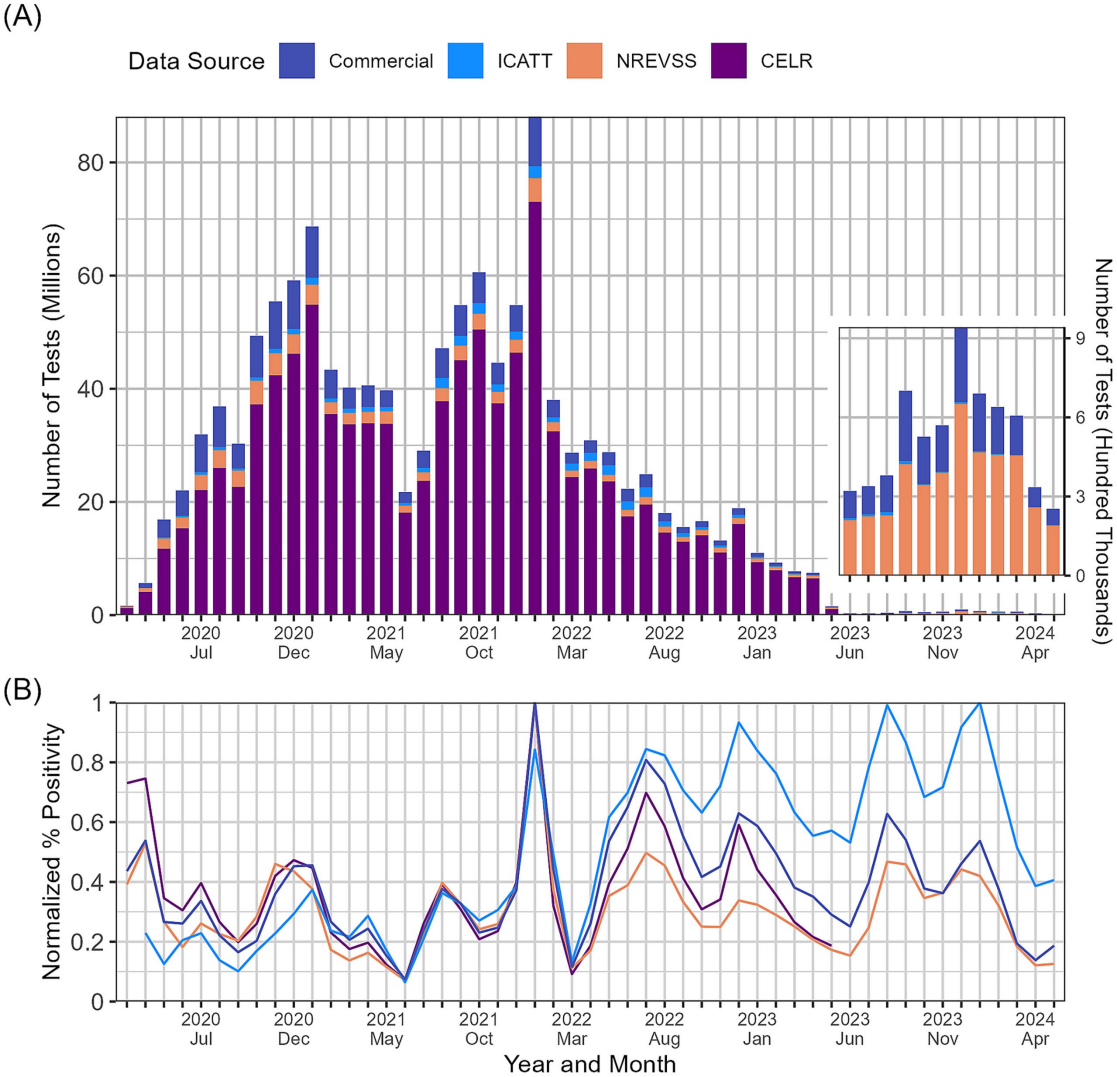
National monthly normalized percent positivity and SARS-CoV-2 test volume over time by laboratory-based COVID-19 surveillance data source, 2020–2024. **(A)** The testing volumes for months are depicted. The bars are analogously colored to represent the total testing volume according to each data source for each month. The CELR bar encompasses the other bars to indicate that CELR contains data from the other data sources. The x axis is shared with part B. June 2023 through May 2024 are shown in the inset graph to show greater detail about testing volumes during this time. **(B)** Monthly percent positivity was normalized to the maximum percent positivity of each data source and depicted. Line colors indicate the various data sources, and the values indicate the monthly normalized percent positivity. Abbreviations: COVID-19 electronic laboratory reporting (CELR); Increasing Community Access to Testing, Treatment, and Response (ICATT); National Respiratory and Enteric Virus Surveillance System (NREVSS).

Overall, SARS-CoV-2 testing volume largely decreased over the entire evaluation period among all data sources (Figure 1). CELR testing volume peaked during early January 2022 at 20,369,462 weekly tests. NREVSS testing volume peaked in late November 2020 at about 19-fold fewer weekly tests (1,094,536) compared to CELR. ICATT peaked in late August 2021, with about 36-fold fewer tests (559,865) compared to CELR. Commercial laboratory testing volume also peaked in late November 2020 (like NREVSS) but with over eight-fold fewer weekly tests (2,402,864) compared to CELR. There has been a substantial drop in laboratory testing volume since January 2022 across all data sources, especially for CELR data.

Analysis of the monthly number of HSAs covered by each dataset showed that NREVSS data represented a small, consistent set of reporters with a median of 173 HSAs represented each month (IQR: 9) out of 3,436 total HSAs. In comparison, commercial laboratories (median = 713, IQR: 343), ICATT (median = 1,236.5, IQR: 1,375), and CELR (median = 929, IQR: 18) had a higher number of HSAs represented. Overall, CELR had the lowest CQV (0.02), followed by NREVSS (CQV = 0.05), commercial laboratories (CQV = 0.5), and ICATT (CQV = 1.1).

### 3.2 Viral activity

**Figure 2:**
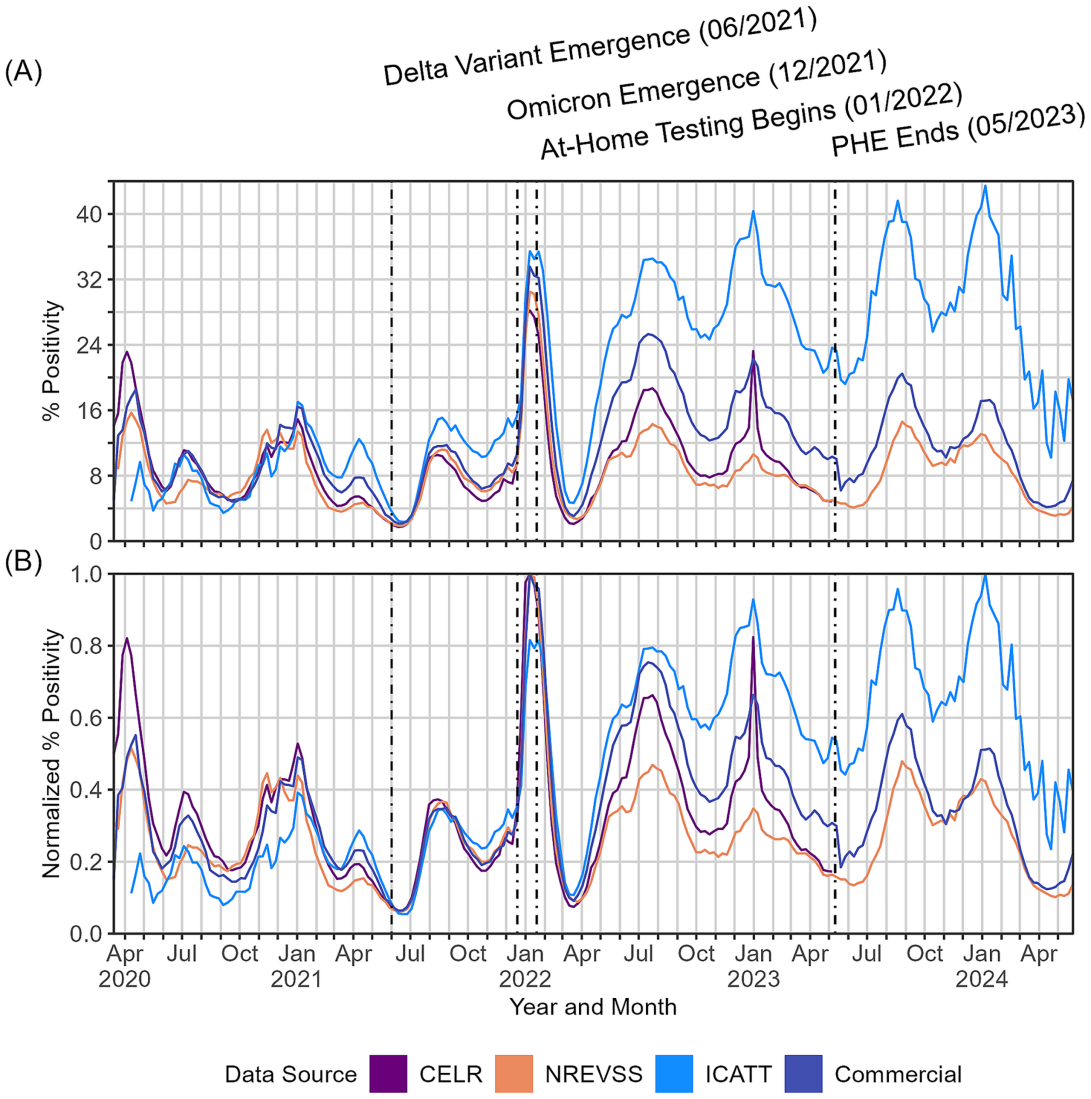
Weekly percent positivity and normalized weekly percent positivity with key events over time by laboratory-based COVID-19 surveillance data source, 2020–2024. Weekly percent positivity **(A)** is shown along with normalized weekly percent positivity **(B)**. The percent positivity is normalized to the maximum percent positivity of each data source, so that the maximum of each data source is 1.0. Key events are marked with a dashed line. The date labels on the dashed lines refer to the date when the event occurred. Key events include the SARS-CoV-2 Delta variant attaining U.S. predominance (≥50% estimated prevalence) on June 1, 2021; the Omicron variant attaining U.S. predominance on December 19, 2021; at-home testing kits becoming available for order online from the U.S. government on January 19, 2022; and the end of the COVID-19 public health emergency on May 11, 2023. Abbreviations: COVID-19 electronic laboratory reporting (CELR); Increasing Community Access to Testing, Treatment, and Response (ICATT); National Respiratory and Enteric Virus Surveillance System (NREVSS).

Median weekly percent positivity in CELR (8.4%) and especially NREVSS (7.7%) tended to be lower overall compared with ICATT (18.5%) and commercial laboratory (11.0%) data (Table 2). The Wilcoxon sign-rank test suggested that weekly percent positivity for all data sources were significantly greater than NREVSS overall and during any key period (*p* < 0.05). Median weekly percent positivity within all data sources increased after the emergence of the Delta variant and further increased in the post-Omicron period, which coincided with availability of at-home testing starting in January 2022. Differences in median weekly percent positivity between the data sources widened substantially after March 2022 (Figures 1, 2).

**Table 2:** Median (IQR) weekly percent positivity by laboratory-based COVID-19 surveillance data source and by key event period, 2020–2024.*

Key event period	COVID-19 Electronic Laboratory Reporting (CELR)	National Respiratory and Enteric Virus Surveillance System (NREVSS)**	Increasing Community Access to Testing, Treatment, and Response (ICATT)	Commercial laboratories
Overall	8.4% (6.1)	7.7% (5.5)	18.5% (19.9)	11.0% (8.4)
Pre-Delta variant	7.6% (6.2)	6.6% (5.9)	8.3% (5.1)	7.8% (5.7)
Post-Delta variant	8.6% (6.3)	8.2% (5.0)	25.9% (16.7)	11.6% (8.9)
Pre-Omicron variant	7.2% (5.2)	7.0% (4.8)	9.3% (5.8)	7.9% (4.9)
Post-Omicron variant	9.8% (7.0)	8.4% (5.4)	27.7% (11.4)	13.5% (8.1)
Before at-home testing	7.5% (5.3)	7.1% (5.2)	9.5% (6.3)	8.0% (5.2)
After at-home testing	9.4% (6.6)	8.4% (5.3)	27.6% (11.1)	13.0% (7.8)
Before end of public health emergency	8.4% (6.1)	7.4% (4.7)	12.8% (18.5)	11.0% (8.8)
After end of public health emergency	N/A	9.2% (6.9)	28.9% (15.3)	11.4% (7.6)

* Median (interquartile range, IQR = Q3–Q1) weekly percent positivity was calculated by data source (CELR, NREVSS, ICATT, and commercial laboratories) for key event periods. A key event period is defined as either the period before or after key events relevant to the COVID-19 pandemic, when data were available. **Overall and during each key event period, weekly percent positivity was significantly greater (p < 0.05) in all other data sources when compared to NREVSS (Wilcoxon signed-rank test).

### 3.3 Viral activity trends

Overall, the correlations of normalized SARS-CoV-2 percent positivity for NREVSS vs. CELR (*ρ* = 0.93) and NREVSS vs. commercial laboratory data (*ρ* = 0.85) were high, indicating consistent viral activity trends (Table 3). The specific correlations for NREVSS vs. CELR percent positivity remained relatively unchanged across all evaluated comparison periods (*ρ* > 0.90). The correlations for NREVSS vs. commercial laboratory data were higher after the end of the PHE (*ρ* = 0.93) than before (*ρ* = 0.83) and higher post-Delta (*ρ* = 0.86) than pre-Delta (*ρ* = 0.77). These correlations were relatively unchanged before and after the emergence of Omicron (*ρ* = 0.85 vs. 0.88, respectively) and before and after the availability of at-home testing (*ρ* = 0.87 vs. 0.88). NREVSS vs. ICATT overall had a lower correlation (*ρ* = 0.58). Prior to the Delta period, the correlation for NREVSS vs. ICATT was low (*ρ* = 0.25) but correlations were higher after this period. Each pre-period had a lower correlation for NREVSS vs. ICATT than the corresponding post-period (Delta: *ρ* = 0.25 vs. 0.72; Omicron: *ρ* = 0.54 vs. 0.80, at-home testing: *ρ* = 0.60 vs. 0.83, PHE: *ρ* = 0.60 vs. 0.91).

**Table 3:** Correlations between NREVSS and other laboratory-based COVID-19 surveillance data sources within key event periods, 2020–2024*.

Key event period	NREVSS vs CELR	NREVSS vs ICATT	NREVSS vs commercial
Overall	0.93^++^	0.58^++^	0.85^++^
Pre-Delta variant	0.93^++^	0.25^++^	0.77^++^
Post-Delta variant	0.94^++^	0.72^++^	0.86^++^
Pre-Omicron variant	0.90^++^	0.54^++^	0.85^++^
Post-Omicron variant	0.96^++^	0.80^++^	0.88^++^
Before at-home testing	0.92^++^	0.60^++^	0.87^++^
After at-home testing	0.95^++^	0.83^++^	0.88^++^
Before end of public health emergency	0.93^++^	0.60^++^	0.83^++^
After end of public health emergency	N/A	0.91^++^	0.93^++^

COVID-19 electronic laboratory reporting (CELR); Increasing Community Access to Testing, Treatment, and Response (ICATT); National Respiratory and Enteric Virus Surveillance System (NREVSS).

* Spearman’s correlation coefficient (*ρ*) was calculated between NREVSS and each of the other data sources (CELR, ICATT, commercial) for each comparison period. A comparison period is demarcated by COVID-19 pandemic related key events, for time when data were present for both sources. In the table, “++” indicates *p* ≤ 0.05, where *p* was calculated using a *t*-test to determine whether the correlation was significantly different from 0.

**Figure 3:**
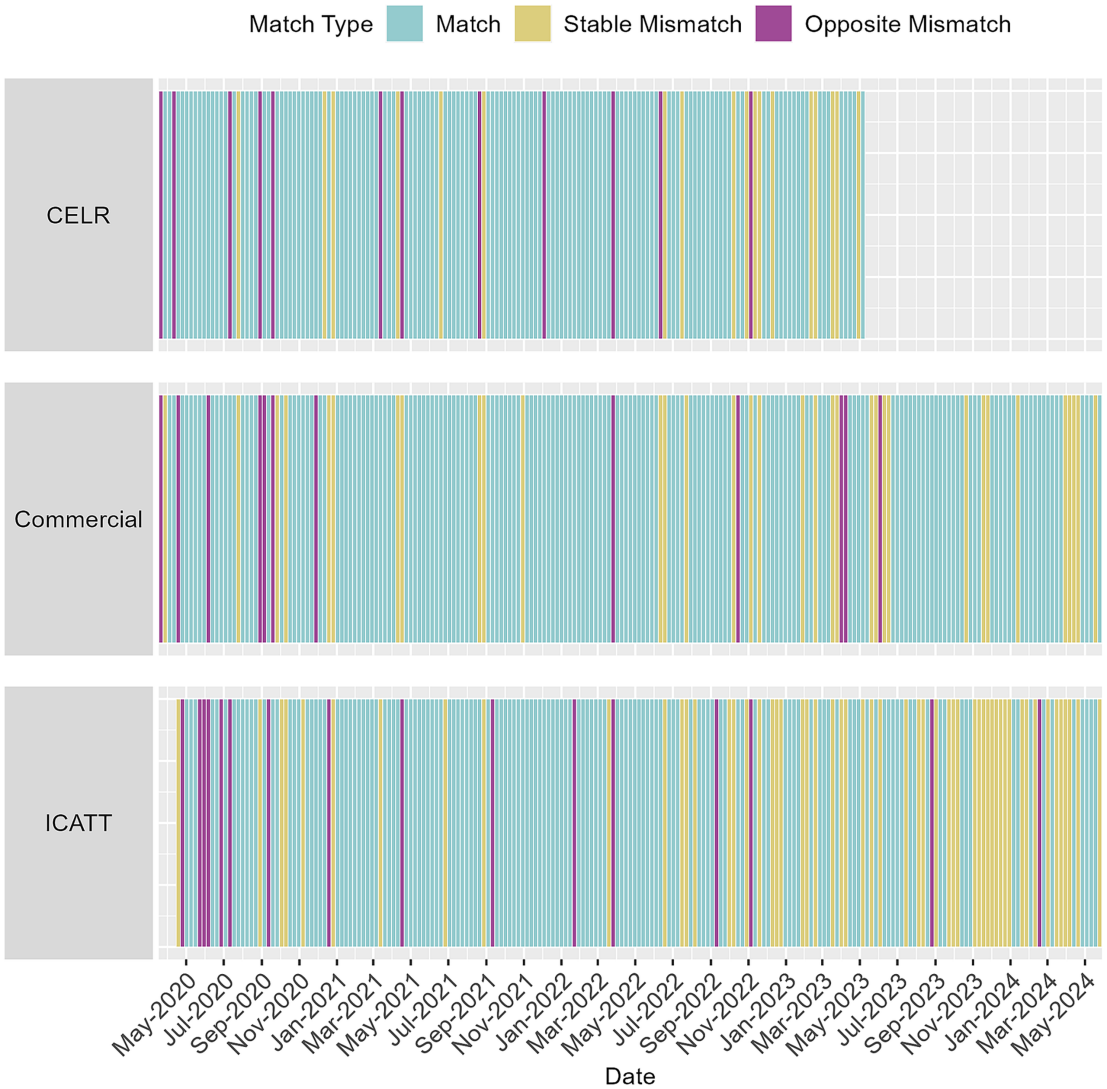
Trajectory matching comparing NREVSS and other laboratory-based COVID-19 surveillance data sources, 2020–2024. Weekly change in percent positivity was calculated by subtracting the previous week percent positivity from the current week percent positivity. The standard error of the change in percent positivity was calculated, and a 95% confidence interval was determined using +/− 1.96 * SE. Depending on whether the weekly change in percent positivity was outside or inside the confidence interval, the weekly change in percent positivity was categorized as ‘Stable’ or ‘Not Stable’, respectively. ‘Not Stable’ weeks were further categorized as either an increase or a decrease depending on whether the change was positive or negative. For each week the categorizations were compared between NREVSS and the other data sources and evaluated on being a match (in agreement), stable mismatch (one data source indicated stable while the other did not), or opposite match (one data source said increase while the other said decrease). The rows in the figure indicate which data source was being compared against NREVSS. The X axis indicates the week being compared. The bars are colored according to the results of the evaluation and indicate a match, stable mismatch, or opposite match. Grey spaces indicate time when data were not available in both NREVSS and the data source of comparison. Abbreviations: COVID-19 electronic laboratory reporting (CELR); Increasing Community Access to Testing, Treatment, and Response (ICATT); National Respiratory and Enteric Virus Surveillance System (NREVSS).,

The trajectory matching analysis showed that, despite differences in the magnitude of estimated viral activity between data sources, overall week-to-week trajectories were very similar when compared to NREVSS (Figure 3). Overall, 134 (82%) of 164 weeks had the same trajectory for NREVSS vs. CELR. 173 (79%) of 219 weeks had the same trajectories for NREVSS vs. commercial laboratory data, and 143 (67%) of 215 weeks were the same in NREVSS vs. ICATT.

### 3.4 Sample overlap of CELR and other data sources

Between CELR and NREVSS, sample overlap varied between 23% in the early evaluation period and 46% in the late evaluation period. Between CELR and commercial, sample overlap varied between 55% in the early evaluation period and 20% in the late evaluation period. Between CELR and ICATT, sample overlap varied between 2% in the middle of the evaluation period and 0.3% in the late evaluation period.

## 4 Discussion

SARS-CoV-2 testing practices and COVID-19 reporting changed substantially during 2020–2024 based on this evaluation of laboratory-based surveillance data sources. Significant decreases in testing volume have occurred since January 2022. Furthermore, we observed consistent differences in normalized weekly percent positivity indicating that some data sources tended to report higher viral activity levels than others. These differences may reflect testing and care-seeking differences in the underlying populations of each dataset and translate to different probabilities of being positive prior to taking a test. This could also be attributable to the effects of varying reporting practices over time. In contrast, the observed high correlations of CELR, ICATT, and commercial laboratory data sources with NREVSS coupled with few differences in percent positivity trajectories are partially attributable to the sample overlap in data sources, but also suggest that viral activity trends were largely consistent across the data sources.

The observed decline in overall laboratory test volume from January 2022 to May 2023 demonstrates a broader shift in testing practices. Reduced NAAT testing resulted from increased use of SARS-CoV-2 antigen self-tests and foregoing testing altogether due to reduced disease severity, pandemic fatigue, and perceptions of COVID-19 becoming endemic (21–26). Antigen-based tests are less likely to be reported and were excluded from analysis for this evaluation (24, 27). While antigen-based self-tests were available prior to January 2022, uptake increased after tests were made available through a U.S. government website on January 19, 2022 (12, 18). Testing volume in the CELR system declined during this time and some CELR reporters began reporting only positive tests before the end of the PHE declaration. As a result of this change in testing volume, measures of positivity and detections became too unreliable for inclusion in the full evaluation period. In contrast, all other data sources continued reporting positive and negative testing data (e.g., pharmacies contracted under the ICATT program were obligated to report), and the percent positivity data were reliable during the full evaluation period.

Trends in SARS-CoV-2 activity levels captured by laboratory-based surveillance may reflect changing circulation patterns associated with COVID-19 periodicity and the emergence of SARS-CoV-2 variants that substantially altered the incidence of infection (28). However, some variations over time are also likely attributable to artifactual changes in data collection and testing practices within each system. In particular, the divergence in test positivity across data sources after March 2022 coincided with the increased availability of at-home SARS-CoV-2 antigen testing, which reduced laboratory testing volume and impacted percent positivity measures differentially (24). Across key event periods, viral activity was most comparable between NREVSS and CELR, as measured by median weekly percent positivity. NREVSS remained a consistent source of laboratory-based surveillance data after the expiration of the PHE declaration for COVID-19. Median percent positivity in NREVSS and CELR were less variable compared to the other data sources, based on the smaller interquartile ranges (IQR) associated with the median percent positivity estimates. This finding may be related to how NREVSS and CELR have smaller CVQs, which is indicative of reporting from a consistent number of HSAs. In comparison, data from commercial laboratories and ICATT had greater numbers of HSAs represented and were more sensitive to changes in underlying populations. ICATT had a greater median weekly percent positivity and larger corresponding IQR as it initially focused on symptomatic or exposed people, then transitioned to focusing on uninsured people in socially vulnerable communities at high risk for SARS-Cov-2 infection (12). In contrast, NREVSS includes data from all patients who undergo COVID-19 testing in commercial, clinical, and public health laboratories, representing a wide range of hospital-based testing performed for many reasons (9). Consequently, a proportion of NREVSS tests were less likely to be positive and lead to lower percent positivity overall. While commercial laboratories also may include some asymptomatic testing, this proportion varied over time, as testing was initially focused on symptomatic individuals early in the pandemic and transitioned toward asymptomatic testing (29).

While absolute differences in viral activity persisted across data sources after March 2022, normalized percent positivity correlations with NREVSS remained mostly high. Correlations for NREVSS vs. CELR and NREVSS vs. commercial laboratory testing data also fluctuated minimally across key events. Correlations for NREVSS vs. ICATT were lower, but this finding appears to be limited to the earlier part of the evaluation period (March 2020–April 2021). Unlike NREVSS, CELR, and commercial laboratory testing data sources, ICATT was a new program that did not have existing infrastructure to build upon. During its startup period (until approximately April 2021), pharmacy sites were still rapidly joining ICATT, and the sample of people included may not have been representative of the eventual underlying target population (11). Correlations after this period (i.e., post-periods in the analyses of key events) are similar to corresponding correlations from the other data sources. Additionally, all data sources had high match rates according to the trajectory analysis. Though the NREVSS vs. ICATT match rate is lower than the other two data sources, most opposite mismatches for this data source occurred during the same startup period. Taken together, both the correlations and trajectory analysis suggest that the relative differences in viral activity trends between data sources were minimal despite absolute differences in viral activity.

A limitation of this evaluation is that test result data may have been reported to multiple data sources, inflating correlations due to sample overlap. In particular, CELR likely contains data from all other data sources and estimates range from 55 to 0.34% overlap through the course of the pandemic and across data sources Despite this, given CELR’s function as a repository for laboratory-based COVID-19 testing data prior to the expiration of the PHE declaration, it served as a valuable data source for comparison (7, 8, 30). The overlap between NREVSS and commercial laboratories and ICATT could not be quantified due to the structure of the data. The commercial laboratory dataset may include testing data also captured in NREVSS, particularly during periods of high testing volume, as commercial laboratories are sometimes utilized by hospitals for additional testing capacity (27). Pharmacies also may use commercial laboratories in a similar manner, suggesting potential overlap between ICATT and commercial laboratory data. While the tests populations are not distinct across data sources, overlap is only expected to occur in certain parts of the country where the catchment areas of NREVSS reporters or ICATT pharmacies overlap with commercial laboratory service locations. This characteristic is more likely in rural areas where hospital laboratory capacity is limited and fewer tests are performed (27, 31).

Regarding match rates, the independence assumption is not met with people who are likely to be tested from week to week. As a result, the standard errors and confidence intervals may be underestimated and narrower, respectively. Ultimately, more weeks may be characterized as an increase or decrease when they are stable. This affects the sensitivity of the metric, but we do not expect independence to vary by data source. Repeat testing leading to non-independence was likely more prevalent during periods of high transmission when policies such as mandated testing were implemented non-differentially on each of the data source populations. Match rates may undercount stable mismatches and overcount opposite trajectory mismatches. Since these are both summed up to find the matching trajectory proportions, the proportion would be unaffected.

Duplicated reporting within a data source is another limitation. No specimen-level data were available to enable deduplication.

Due to most laboratories initially conducting NAAT-based testing, and the growing use of multiplex testing (e.g., SARS-CoV-2, influenza, and respiratory syncytial virus), this analysis excluded antigen-based testing (32, 33). In the ICATT program, antigen testing became more prevalent over the course of the pandemic as it was more cost effective (12). ICATT laboratory NAAT testing volume in this analysis became very low in the last year (May 2023–May 2024); however, these data were included for completeness, and trends still generally appeared to align with other data sources. Although self-testing results that employ rapid antigen-based methods have constituted a larger portion of all COVID-19 testing since 2022, antigen test data have lower sensitivity relative to NAATs (34–36). The main goal of this evaluation was to establish an understanding of the relative strengths of data captured by existing laboratory-based COVID-19 surveillance systems. Self-test data were excluded, as they are not routinely reported and require post-pandemic infrastructure investments to be fully utilized as surveillance data sources in the future (34, 37).

This evaluation focused on describing the reliability and concordance of laboratory-based surveillance, which is one component of integrated systems that collectively have multiple applications (38). Together, these systems provide a public health foundation for genomic surveillance, epidemiological modelling, and advanced analytics that inform evidence-based containment policies (28, 39–41).

## 5 Conclusion

Absolute differences in viral activity, indicated by SARS-CoV-2 testing data reported among various surveillance data sources, changed substantially during the evaluation period. Despite these differences and reduction in testing volume over time, relative differences in viral activity trends were minimal. The correlations in viral activity trends across data sources and consistency in weekly trajectories between NREVSS and other data sources suggest concordance in trends, though data overlap has a role in this relationship. High match proportions across all data source comparisons indicated that laboratory-based surveillance data from different sources can be used in parallel to track COVID-19 activity trends reliably. These findings highlight that a broad set of robust laboratory-based surveillance data sources captured trends in SARS-CoV-2 circulation effectively at the national level despite the evolving nature of testing and reporting during and after the COVID-19 pandemic.

## Data Availability

The NREVSS and CELR datasets analyzed in this study can be found at (1, 2), respectively. Commercial laboratory data analyzed in this study are currently confidential but may be available in the future on the One CDC Data Platform (3). ICATT data are confidential. 1: [https://data.cdc.gov/Laboratory-Surveillance/Percent-Positivity-of-COVID-19-Nucleic-Acid-Amplif/gvsb-yw6g/about_data] 2: [https://healthdata.gov/dataset/COVID-19-Diagnostic-Laboratory-Testing-PCR-Testing/j8mb-icvb/about_data] 3: [https://www.cdc.gov/nssp/php/partnerships/cdc-streamlines-access-to-commercial-laboratory-data.html].

## Footnotes

1 See, e.g., 45C.F.R. part 46.102(l) (2), 21C.F.R. part 56; 42 U.S.C. §241(d); 5 U.S.C. §552a; 44 U.S.C. §3,501 et seq.
